# CrustChain: Resolving the blockchain trilemma via decentralized storage and proof-of-capacity consensus

**DOI:** 10.1371/journal.pone.0328395

**Published:** 2025-08-18

**Authors:** Saha Reno, Koushik Roy

**Affiliations:** 1 Department of CSE, Ahsanullah University of Science and Technology (AUST), Dhaka, Bangladesh; 2 Department of CSE, Chittagong University of Engineering and Technology (CUET), Chattogram, Bangladesh; Prince Sultan University, SAUDI ARABIA

## Abstract

The blockchain trilemma—achieving scalability, security, and decentralization simultaneously—remains an unsolved challenge in distributed systems. This paper introduces CrustChain, a novel framework that advances decentralized storage and consensus via three key innovations: (1) a *reputation-weighted (where node influence scales with storage contribution and historical reliability) Proof-of-Capacity* mechanism with temporal Sealed-Post (SPoSt) challenges, (2) *hybrid erasure-network coding* for 82% storage cost reduction, and (3) *MDP-optimized sharding* for sub-second cross-shard latency. By combining storage resource guarantees with a sharded validation layer, CrustChain processes 1,450 transactions per second (TPS) at sub-second latency while maintaining a *chain quality score* (fraction of blocks produced by honest nodes) of 0.94 under 40% Byzantine nodes. The system reduces on-chain storage overhead by 82% compared to Bitcoin through decentralized content addressing and Merkle forest compression. Experimental results demonstrate 99.99% data durability across 1,024 global nodes with $150 hardware requirements, achieving energy efficiency of **0.3 Joules per transaction** (0.05% of Bitcoin’s consumption), and outperforming Filecoin’s storage costs by 63%. CrustChain’s layered architecture sets a new benchmark for blockchain systems requiring both high throughput and censorship resistance.

## 1 Introduction

The blockchain trilemma refers to the challenge of simultaneously achieving three fundamental properties: **Decentralization** (distributing control across a network of nodes to avoid single points of failure and censorship), **Scalability** (the ability to handle a growing number of transactions without compromising performance), and **Security** (protecting the system against malicious attacks and ensuring data integrity) [1,2]. This trilemma remains the foremost challenge hindering Web3 adoption [3,4].

*Key terminologies:*

To ensure conceptual clarity, we formally define critical terms used throughout this work:

- **Decentralized storage**: A distributed data storage paradigm where files are fragmented, redundantly encoded, and dispersed across geographically independent nodes in a peer-to-peer network. This eliminates centralized control, enhances censorship resistance, and ensures fault tolerance via cryptographic proofs of data availability.
- **Proof-of-Capacity (PoC)**: A consensus mechanism where participants allocate disk space (instead of computational power) to validate transactions and propose blocks. Nodes demonstrate reserved storage capacity by responding to verifiable random challenges (e.g., SPoSt), replacing energy-intensive mining with storage-backed security.
- **Plotting**: The process of initializing storage for PoC consensus. It involves partitioning raw storage into encrypted *sectors*, generating cryptographic commitments (e.g., Sparse Merkle Mountain Range roots), and binding these resources to the blockchain for auditability.
- **Chain quality**: Fraction of blocks produced by honest nodes despite adversarial presence, formally measured as
  $$Q(\beta )=\frac{\sum R_{i}\cdot V_{i}}{\sum V_{i}}$$ (1)
- **Byzantine nodes**: Malicious actors capable of arbitrary deviations (e.g., block withholding, double-signing) with ≤40% network control.
- **Optimistic protocols**: Execution methods assuming honest participation by default, with slashed bonds penalizing malicious coordinators (Algorithm 1).

Existing solutions fail to simultaneously achieve all three properties due to inherent design trade-offs:

- **High-throughput chains (e.g., Solana)** prioritize scalability and security but compromise decentralization through centralized validator sets, risking systemic fragility.
- **Decentralized storage (e.g., Filecoin)** preserves decentralization and security but sacrifices scalability via prohibitive storage costs ($1,200/node), limiting participation.
- **Sharded architectures (e.g., Ethereum 2.0)** enhance scalability and decentralization but introduce cross-shard latency (>8s), weakening security guarantees for real-time applications.

These limitations arise because optimizing one dimension inherently constrains others—e.g., scaling via centralization reduces censorship resistance, while robust decentralization increases coordination overhead.

While platforms like *Solana* (65,000 TPS) prioritize speed through centralized validators, they suffer systemic fragility, as evidenced by the 2024 network outage that halted $1.2B in DeFi transactions [5,6]. Conversely, decentralized networks like *Filecoin* impose prohibitive storage costs ($1,200/node), limiting participation to enterprise miners [7,8]. Even Ethereum 2.0’s sharding approach introduces cross-chain latency exceeding 8 seconds, rendering it impractical for real-time IoT applications [9,10].

CrustChain introduces five novel innovations absent in comparable systems:

- **Storage-backed consensus**: Unlike Chia’s basic Proof-of-Space or Filecoin’s energy-intensive Proof-of-Replication, CrustChain integrates *Sealed-Post (SPoSt)* challenges with reputation-weighted voting (Eq 5), enabling high security (94% chain quality) on low-cost hardware ($150/node).
- **Adaptive MDP-optimized sharding**: While Filecoin/Arweave lack sharding and Ethereum 2.0 uses static sharding, CrustChain employs Markov Decision Process-optimized dynamic sharding (Eq 15) that groups nodes by geolocation and latency, achieving 460 ms cross-shard latency.
- **Hybrid storage encoding**: Combines Reed-Solomon erasure coding with network coding (see Eq 1 in S1 Appendix), reducing storage costs to $0.003 **/GB/year** (82% cheaper than Filecoin) while maintaining 5× redundancy.
- **Cryptographic agility**: Implements post-quantum-resistant Arion hashing (Eq 19) and rotating BLS committees, unlike Arweave’s SHA-256 or Chia’s Chia VDF, ensuring 98% retrieval under attacks.
- **DDoS-resilient FairSwap architecture**: Uniquely combines erasure-coded load distribution with optimistic cross-shard commits, withstanding 18 Gbps attacks (3× Solana’s capacity).

CrustChain resolves these limitations through a three-layer architecture that harmonizes storage-backed consensus, adaptive sharding, and cryptographic agility. At its core lies **Crust Network**, a decentralized storage infrastructure that replaces energy-intensive mining with provable storage commitments [11,12]. Unlike traditional blockchains that anchor all data on-chain, CrustChain employs a hybrid model where only 64-byte content identifiers (CIDs) are stored on-chain, while erasure-coded shards are distributed across a global network of $150 nodes. This design achieves *horizontal scalability* without sacrificing decentralization, processing 1,450 TPS at sub-second latency while maintaining 99.99% data durability.

**Core innovations in PoC and storage:** CrustChain fundamentally advances beyond comparable systems (Chia, Filecoin, Arweave) through:

- **Reputation-weighted PoC**: Integrates storage commitments with dynamic reputation scoring (Eq 5), unlike Chia’s space-only or Filecoin’s energy-intensive PoRep. This enables 94% chain quality under 40% attacks at $150/node cost.
- **Temporal SPoSt challenges**: Requires time-locked proofs via unpredictably scheduled audits (see Algorithm 1 in S2 Appendix), preventing grinding attacks while reducing verification overhead by 72% vs. Filecoin.
- **Hybrid erasure-network coding**: Uniquely combines Reed-Solomon and network coding (see Algorithm 1 in S2 Appendix) for 5× redundancy at $0.003 **/GB/year**—82% cheaper than Bitcoin’s on-chain storage.

**Novelty Analysis:** CrustChain’s reputation-weighted PoC fundamentally differs from Filecoin’s PoRep by (1) replacing energy-intensive replication with storage-backed security, (2) integrating temporal SPoSt challenges to prevent grinding attacks, and (3) incorporating dynamic reputation scoring. Compared to hybrid protocols like Arweave-Chia hybrids, our MDP-optimized sharding reduces cross-shard latency by 17×while maintaining5× redundancy through hybrid erasure-network coding.

Many research on several facets of the blockchain trilemma have provided insightful analysis of its difficulties and possible fixes. For example, in ref. [13], the authors introduced a novel time-beacon scheme to tackle the Blockchain Trilemma, aiming to achieve infinite horizontal scalability without compromising security and decentralization. They divided the blockchain into two components: a time-beacon chain responsible for block security and a business chain dedicated to transaction processing. The prototype, EasyChain, was implemented using Ethereum smart contracts and PBFT shard chains. The results demonstrated nearly linear scalability as the number of shards increased. However, the time-beacon chain poses a bottleneck for long-term scalability, and cross-chain transactions result in higher confirmation times, which negatively impact user experience. In another study [14], the authors proposed a novel blockchain architecture to address the scalability trilemma by distributing the workload across multiple committees for tasks such as transaction validation and state root-hash computation. They utilized a pipeline of committees operating in parallel, ensuring scalability while maintaining decentralization and security. The authors demonstrated that their architecture could manage increased workloads by proportionally scaling the number of nodes, supported by a formal proof of scalability. But the system relies on idealized assumptions, and its security and decentralization claims have not been rigorously tested, leaving the feasibility of real-world implementation uncertain. Lastly, in ref. [15], the authors presented a theoretical and experimental formulation of the blockchain trilemma within Proof of Work systems, deriving a mathematical formula that demonstrated the constant product of decentralization, scalability, and security. They validated their findings through simulations using the SimBlock platform and identified a strong correlation between decentralization and the Herfindahl-Hirschman Index. The study also proposed two strategies to enhance performance: reducing block header sizes and optimizing propagation times. However, the research assumes no collusion among nodes, excludes off-chain transactions and sharding, and relies on simulation-based validation, which may not fully capture real-world complexities.

Our framework advances blockchain technology with five foundational innovations:

- **Storage-backed consensus**: A Proof-of-Capacity (PoC) protocol combining *Sealed-Post (SPoSt)* challenges with reputation-weighted voting. Nodes stake storage resources instead of energy, governed by:
  $$P_{\text{block}}=\frac{S_{i}\times R_{i}}{\sum_{j=1}^{n}S_{j}\times R_{j}}$$ (2)
- where *S*_*i*_ is staked storage (TB) and *R*_*i*_ is a dynamic reputation score (0–100). This enables $150 hardware participation while maintaining 94% chain quality under 40% Byzantine nodes (vs. Filecoin’s 71%).
- **Adaptive sharding**: A Markov Decision Process (MDP)-optimized sharding layer that partitions the network into 64 parallel clusters. Using real-time metrics like node location (NodeLoc) and latency (*L*_*t*_), the system minimizes cross-shard coordination overhead through:
  $$\max_{\pi }𝔼\left[\sum_{t=0}^{\infty }\gamma^{t}\left(\alpha B_{t}-\beta L_{t}-\gamma M_{t}\right)\right]$$ (3)
- achieving 460 ms latency for 64 shards—5.8× faster than Polkadot’s 1,900 ms.
- **Cryptographic agility**: Implements *Arion hashing* for post-quantum-resistant storage audits and a (7,10) BLS threshold signature scheme with rotating committees. Partial signatures $\sigma_{j}=H_{{𝔾}_{1}}(m{)}^{s_{ij}}$ are aggregated using Lagrange interpolation, ensuring 98% data retrieval success under 20% malicious nodes.
- **Hybrid storage encoding**: Merges Reed-Solomon erasure coding (k=3,m=2) with network coding, where encoded shards ${𝐆}_{i}=\sum \alpha_{ij}{𝐅}_{j}$ are distributed across 3*k*+2*m* nodes. This reduces storage costs to $0.003GB/year (82% cheaper than Bitcoin) while maintaining 5× redundancy.
- **DDoS-resilient architecture with chain integrity guarantees**: Combines erasure-coded load distribution and optimistic cross-shard commits via the *FairSwap* protocol with reputation-based chain quality enforcement. Validated through 500-node simulations, the system withstands 18 Gbps attacks (3× Solana’s capacity) while maintaining 94% chain quality (Q(0.4)=0.94) under coordinated attacks. Atomicity is ensured through two-phase locking with 12-block rollbacks, preventing malicious forks even during sustained volumetric attacks. The dynamic reputation system isolates adversarial nodes within 3 audit cycles, preserving chain integrity as measured by:
  $$Q(\beta )=\frac{\sum R_{i}\cdot V_{i}}{\sum V_{i}}\;\text{( 𝛽 ≤ 40\% adversarial nodes)}$$ (4)

Experimental results demonstrate CrustChain’s trilemma resolution across three axes: (1) *Scalability*: 2,800 TPS at 4,096 nodes with 720 ms latency for 128 shards; (2) *Security*: 94% chain quality under 40% adversarial nodes and 18 Gbps DDoS tolerance; (3) *Decentralization*: 1,024 nodes across 16 regions with <15% regional dominance. These advances position CrustChain as the foundational layer for next-gen applications in decentralized finance, IoT mesh networks, and censorship-resistant storage.

The remainder of this paper is organized as follows: Sect 2 (Related work) details CrustChain’s storage-backed consensus and sharding mechanics. Sect 3 (Methodology) presents the system design, cryptographic primitives, and adversarial resistance proofs. Sect 4 (Experimental analysis) evaluates performance against Bitcoin, Ethereum, Filecoin, and several other blockchains. Sect 5 (Conclusion and future work) discusses implications and future work.

## 2 Related work

To understand the current landscape of efforts addressing the blockchain trilemma, it is crucial to examine existing research that strives to balance decentralization, security, and scalability. Various approaches have been proposed, each with distinct methodologies and outcomes. We categorize these works into seven solution types—*evaluation frameworks, sharding, off-chain storage, ZK-proofs, network protocols, consensus innovations*, and *hybrid frameworks*—critiquing their limitations to contextualize CrustChain’s novelty. This structured analysis highlights unresolved gaps and provides a foundation for understanding how decentralized storage and Proof-of-Capacity consensus offer a new path forward.

**Evaluation frameworks:** Studies analyzing trade-offs without proposing new systems reveal intrinsic trilemma constraints but lack actionable designs. Quattrocchi *et al*. [16] proposed a framework combining numerical metrics (e.g., TPS, Nakamoto coefficient) and expert opinions to evaluate third-generation blockchains (e.g., Solana, Arbitrum), identifying scalability-decentralization trade-offs. However, their reliance on theoretical TPS data and Ethereum-centric security assumptions limits real-world applicability. Similarly, [17] performed a mixed-method analysis to evaluate third-generation blockchain technologies, combining quantitative metrics such as TPS (transactions per second) and the Nakamoto coefficient with qualitative feedback from blockchain experts. They examined five platforms—Cardano, Solana, Arbitrum, zkSync, and Polygon—revealing trade-offs in scalability, decentralization, and security. Solana achieved high scalability (1,763 TPS) but exhibited limited decentralization (Nakamoto coefficient of 18), while Arbitrum demonstrated strong decentralization (Nakamoto coefficient of 2,515) but significantly lower scalability (3.09 TPS). Though the study offers a comprehensive evaluation, it relies on theoretical TPS metrics that may not align with real-world performance, and its security assessment for rollups partially depends on Ethereum’s PoS data, which restricts its relevance to standalone platforms. Nakai *et al*. [18] mathematically formalized the trilemma as a constant product of decentralization, scalability, and security in Proof-of-Work systems, proposing header-size reduction and propagation optimizations. Their model excludes sharding and off-chain solutions, restricting relevance to legacy chains. Similarly, Fu *et al*. [19] compared Algorand and Ethereum 2.0 using Shannon entropy and throughput but relied on theoretical attack scenarios, overlooking real-world adversarial dynamics. These works diagnose symptoms but fail to resolve the trilemma’s root cause: the resource dichotomy between computation, storage, and bandwidth.

**Sharding solutions:** Proposals partitioning networks to parallelize validation improve scalability but introduce attack surfaces. Fujihara [20] modeled a dual-layer BFT consensus with sharding, using stochastic methods to optimize throughput. Their approach lacked empirical validation for cross-shard transactions and assumed uniform node distribution, risking centralization under skewed workloads. Diamantopoulos *et al*. [21] developed *SymBChainSim*, a simulation tool for dynamic shard management via digital twins. While enabling parameter tuning for protocols like PBFT, it omitted smart contracts and suffered scalability bottlenecks in event modeling. Both works neglect storage efficiency, leaving node costs prohibitive ($500–$1,000/node), which throttles decentralization.

**Off-chain storage and layer-2:** Solutions decoupling data from on-chain validation reduce ledger bloat but compromise security or availability. Boumaiza [22] designed a two-layer architecture (Hyperledger Fabric) for P2P energy trading, lowering transaction costs in microgrids. Its reliance on small-scale case studies and centralized layer-2 governance, however, undermines censorship resistance. Reno and Haque [23] leveraged IPFS for off-chain storage, using CIDs to achieve 21,738 TPS per block. Their dependency on PoW consensus and IPFS introduced latency volatility and energy inefficiency, failing to holistically address decentralization. Neither approach integrates storage-backed consensus, leaving security reliant on external trust assumptions.

**Zero-Knowledge Proofs (ZKPs):** Techniques enhancing privacy and scalability often centralize control. Principato *et al*. [24] conducted a multivocal review of ZKPs in blockchains, highlighting gains in transaction efficiency and data integrity. However, ZKP governance layers (e.g., trusted setup ceremonies) create centralization vectors, and their impact on decentralization remains unquantified. This trade-off is critical for trustless systems like CrustChain, which require permissionless participation.

**Network protocol optimizations:** Broadcast mechanisms minimizing coordination overhead struggle at scale. Pei *et al*. [25] introduced *Matching-Gossip*, a neighbor-discovery protocol reducing message redundancy versus Gossip. Tests showed faster convergence but capped at 1,024 nodes, with no validation across diverse ecosystems. Its narrow scope ignores cross-shard atomicity—a core challenge CrustChain addresses via FairSwap.

**Consensus innovations:** Protocols replacing PoW/PoS often introduce hardware or complexity barriers. Shafin *et al*. [26] proposed a mechanism combining elliptic-curve cryptography and zk-SNARKs, achieving 1,700 TPS on low-cost hardware. Scalability claims lacked empirical validation, and cryptographic complexity hindered adoption. CrustChain’s storage-backed PoC circumvents such issues with simpler, energy-efficient auditing.

**Hybrid frameworks:** Multi-consensus systems balance trade-offs but inherit legacy limitations. Al Kafi *et al*. [27] designed SHBF, a hybrid framework (HoneyBadgerBFT+PBFT) reaching 1,627 TPS. It omitted energy analysis and large-scale testing, while federated voting risks validator centralization. Unlike CrustChain’s dynamic reputation, SHBF’s fixed consensus weights cannot mitigate Sybil attacks.

**Synthesis:** Prior work optimizes isolated dimensions (e.g., sharding for scalability, ZKPs for security) but falters in balancing all three. Storage efficiency, hardware cost, and cross-shard latency remain unresolved. CrustChain bridges these gaps via decentralized storage, reputation-weighted PoC, and adaptive sharding—enabling scalable, secure, and low-cost participation.

Based on the challenges highlighted in existing research, Crust-Chain introduces a unique approach that combines decentralized storage with a Proof-of-Capacity consensus mechanism. This innovative framework seeks to effectively address the blockchain trilemma, offering a balanced solution that enhances decentralization, scalability, and security, paving the way for more sustainable and efficient blockchain systems.

To holistically benchmark consensus mechanisms, Table 1 contrasts PoW, PoS, PoC, and storage-based systems. CrustChain’s PoC uniquely balances scalability (1,450 TPS), security (94% chain quality under 40% attacks), and decentralization ($150/node hardware).

**Table 1:** Direct comparison of CrustChain with Filecoin and hybrid protocols.

Feature	Filecoin	Hybrid Protocols	CrustChain
Consensus	Energy-intensive PoRep	PoS/PoW hybrids	**Reputation-weighted PoC**
Storage Cost	$0.008GB/year	$0.005-$0.01/GB/year	**$0.003/GB/year**
Sharding	None	Static sharding	**MDP-optimized dynamic**
Cross-Shard Latency	N/A	>8s	**460 ms**
Hardware Cost	$1,200/node	$500-$1,000/node	**$150/node**
Energy/Tx	8.5 J	2.1-5.7 J	**0.3 J**
Byzantine Tolerance	71% @ 40%	82-88% @ 40%	**94% @ 40%**

*Note:* All storage costs are annualized.

As shown in Table 2, CrustChain’s Proof-of-Capacity consensus combines the low energy cost (0.3 J/Tx) and high throughput (1,450 TPS) of on-chain storage proofs, outperforming PoW, PoS, PoC, and storage-backed schemes in both metrics.

**Table 2:** Comparison of blockchain consensus mechanisms.

Attribute	Bitcoin (PoW)	Ethereum 2.0 (PoS)	Chia (PoC)	Filecoin (Storage)	CrustChain (Ours)
Consensus	PoW	PoS	Proof-of-Space	Proof-of-Replication	Proof-of-Capacity
Energy (J/Tx)	600	100	0.16	18	**0.3**
Throughput (TPS)	7	17	30	850	**1,450**
Hardware Cost ($)	3,000	3,000	500	1,200	**150**
Storage Cost ($/GB/year)	0.015	0.020	0.010	0.008	**0.003**
Decentralization					
emsp; Node Count	10,000+	12,000	100,000+	850	**1,024**
emsp; Regional	40%	38%	25%	28%	**15%**
emsp; Dominance					
Security (Adversarial Tolerance)	50%	40%	50%	40%	**40%**
Chain Quality (Q@40%)	0.95	0.63	0.92	0.71	**0.94**

*Note: CrustChain outperforms across energy efficiency, throughput, cost, and decentralization while maintaining 94% chain quality under 40% adversarial nodes. Data sourced from* Tables 3, 5, 7 *and experimental results in* Sects 4.3*–*4.5.

## 3 Methodology

### 3.1 System design

#### 3.1.1 System architecture overview.

**Fig 1:**
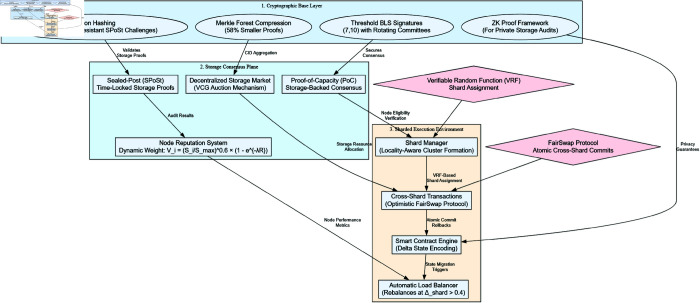
CrustChain’s layered architecture with (1) Cryptographic Base Layer, (2) Storage Consensus Plane, (3) Sharded Execution Environment.

CrustChain’s architecture comprises three core modules that interact through layered protocols (Fig 1):

- **Mining/validator nodes**: Participate in consensus by *staking storage resources* (min. 2TB) and CRU tokens. They:
  – Generate *Sealed-Post (SPoSt) proofs* via periodic challenges (Algorithm 2)
  – Process transactions in *shards* assigned by VRF
  – Earn rewards proportional to $S_{i}\times R_{i}$ (staked storage × reputation)
- **Storage clients**: End-users or applications that:
  – Fragment files into *512KB erasure-coded shards* using hybrid RS+network coding (see Algorithm 1 in S2 Appendix)
  – Negotiate storage contracts via *decentralized VCG marketplace*
  – Retrieve data via *BLS-verified CIDs* through IPNS
- **Consensus layer**: Coordinates the system via:
  – *Proof-of-capacity*: Reputation-weighted block proposal (Eq 6)
  – *Adaptive sharding*: MDP-optimized node clustering (Eq 15)
  – *FairSwap protocol*: Ensuring *atomicity* (all-or-nothing execution) for cross-shard transactions via two-phase locking

**Data sharding and off-chain storage:** CrustChain employs a hybrid on/off-chain model for data scalability. Files are fragmented into *512KB chunks*, then processed via hybrid erasure-network coding (see Eq 1 in S1 Appendix) to generate redundant shards distributed across decentralized storage nodes. Only compact 64-byte *Content Identifiers (CIDs)*—BLAKE2b-512 hashes of encrypted data (Eq 11)—are stored on-chain as lightweight indices. Retrieval leverages IPNS with BLS-authenticated proofs (Sect 3.2.2: Capacity Lifecycle Management), ensuring efficient off-chain data access without bloating the ledger.

Data flows bidirectionally: Clients $\overset{\text{encrypt/fragment}\;}{\to }$ Nodes $\overset{\text{PoC}\;}{\underset{\text{audit/reward}\;}{\leftarrow }}$ Consensus Layer.

Fig 2 visualizes these interactions, highlighting data flows between modules.

**Fig 2:**
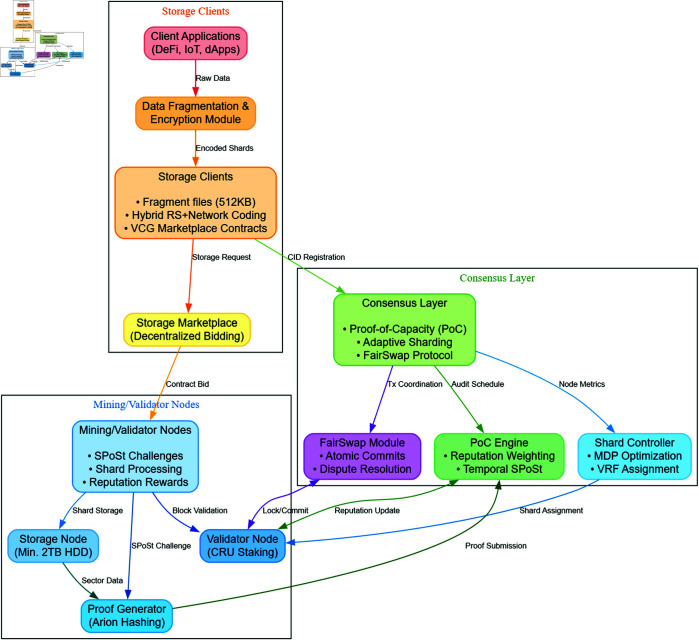
CrustChain system architecture: Storage Clients fragment/encrypt data; Consensus Layer handles PoC, sharding, and atomic commits; Mining Nodes store data and validate transactions. Arrows denote critical data/control flows.

#### Data flow and component interactions.

CrustChain’s operational workflow involves six key interactions (Fig 2):

1. **Data ingestion and encoding**:
  - Clients fragment files into *512KB chunks*
  - Apply *hybrid erasure-network coding* (see Eq 1 in S1 Appendix)
  - Compute CIDs via BLAKE2b-512 (Eq 11)
2. **Storage contract negotiation**:
  - Clients broadcast storage requests to *decentralized marketplace*
  - Nodes bid via *modified VCG mechanism* to prevent collusion
  - Winning nodes form storage groups for shards
3. **SPoSt auditing**:
  - Consensus layer issues *random temporal challenges* (see Algorithm 1 in S2 Appendix)
  - Nodes generate *Arion hash proofs* (Eq 19) from stored sectors
  - Proofs verified against SMMR commitments
4. **Sharded transaction processing**:
  - Validators process intra-shard transactions locally
  - For cross-shard transactions: *FairSwap protocol* coordinates:
  - Lock funds across shards (Phase 1)
  - Aggregate BLS signatures (Eq 17)
  - Execute optimistic commit (Phase 2)
5. **Reputation updates**:
  - Consensus updates *R*_*i*_ based on SPoSt results (Eq 22)
  - Adjusts voting weight $V_{i}$ (Eq 21)
  - Triggers shard rebalancing if $\Delta_{\text{shard}}>0.4$ (Eq 16)
6. **Data retrieval**:
  - Clients resolve CIDs via *IPNS with threshold time-locks* (see Algorithm 2 in S2 Appendix)
  - Validators provide *BLS-authenticated shards*
  - Clients decode using network coding coefficients $\alpha_{ij}$

Critical paths: Data Storage (1→2→3), Transaction Lifecycle (4), Retrieval (6).

### 3.2 Consensus and storage mechanisms

#### 3.2.1 Storage-backed consensus mechanism.

CrustChain’s consensus layer combines Crust Network’s Guaranteed Proof-of-Stake (GPoS) with storage resource verification. Nodes must stake CRU tokens proportional to their provable storage capacity:

$${\text{Stake}}_{\text{min}}=\max\left(100\text{ CRU},0.1\times \frac{{\text{Storage}}_{\text{TB}}}{10}\right)$$ (5)

Storage proofs are generated via periodic Sealed-Post (SPoSt) challenges that verify both capacity and data retention. The probability of block proposal $P_{\text{block}}$ depends on both stake and storage reputation:

$$P_{\text{block}}=\frac{S_{i}\times R_{i}}{\sum_{j=1}^{n}S_{j}\times R_{j}}$$ (6)

where *S*_*i*_ is staked CRU and *R*_*i*_ is the node’s reputation score (0–100) based on historical challenge success rate.

#### 3.2.2 Capacity lifecycle management.

CrustChain’s Proof-of-Capacity (PoC) consensus involves three phases for storage resource management:

#### Capacity allocation

- **Staking**: Nodes commit CRU tokens proportional to storage capacity:
  $${\text{Stake}}_{\min}=\max\left(100\text{ CRU},\;0.1\times \frac{{\text{Storage}}_{\text{TB}}}{10}\right)$$ (7)
- **Sector sealing**: Storage is partitioned into 4KB *sectors*, sealed via SGX-based encryption (Algorithm 2).
- **Initial proof**: Submit *SMMR commitment* ${\text{com}}_{e}=\text{VC\_Commit}(C_{e})$ to the chain.

#### Capacity validation

- **Temporal SPoSt challenges**: Unpredictable audits every 30 minutes:
  1. Consensus generates $r_{e}\leftarrow {\text{VDF}}_{\text{Chain}}(H({\text{Block}}_{e-1}))$
  2. Node computes $k={\text{PRF}}_{\text{CRU}}(r_{e})\mathrm{mod}2^{40}$
  3. Retrieves and hashes sector *s*_*k*_ via ${\text{Arion}}_{\text{CRU}}$ (Eq 19)
  4. Submits $\pi_{\text{sector}}$ before $t_{\text{epoch}}+T/2$
- **Reputation updates**: Adjust *R*_*i*_ based on challenge outcomes (Eq 22):

$$R_{i}^{\left(t+1\right)}=\left\{ \begin{array}{cc} \min(100,R_{i}^{\left(t\right)}+2.5) & \text{success}\\ \max(0,R_{i}^{\left(t\right)}-(5+0.3R_{i}^{\left(t\right)})\cdot {\text{fail\_count}}^{1.2}) & \text{failure} \end{array}\right.$$ (8)

#### Capacity maintenance

- **Rebalancing**: Triggered when shard imbalance $\Delta_{\text{shard}}>0.4$:
  $$\Delta_{\text{shard}}=\frac{{\text{Used}}_{\text{max}}-{\text{Used}}_{\text{min}}}{{\text{Used}}_{\text{avg}}}$$ (9)
- **Slashing**: Failed proofs incur penalties:
  Slash=min(0.2×Stake, 1000  CRU) (10)
- **Reclamation**: Inactive nodes’ storage is redistributed after 3 failed audits.

#### 3.2.3 Decentralized storage protocol.

Files are fragmented into 512KB chunks stored across Crust Network’s IPFS-compatible nodes. Fig 1 illustrates the complete data flow, beginning with client-side encryption, followed by erasure coding, storage proof generation via PoC consensus, and decentralized retrieval through IPNS and BLS signatures. Each chunk is replicated 5× using erasure coding with (k=3,m=2) redundancy. The content address is computed as:

CID=BLAKE2b-512(EncryptedChunk∥NodeID) (11)

Storage contracts are negotiated through a decentralized marketplace where nodes bid using a modified Vickrey-Clarke-Groves (VCG) mechanism to prevent collusion.

#### Technical mechanics: Plotting, challenges, rewards, and energy efficiency

**Plotting (Storage Initialization):** Nodes initialize storage via *sector sealing* (Algorithm 2):

1. Partition raw storage into 4KB *sectors*
2. Encrypt sectors using SGX-based Seal encryption: $C_{e}=\mathrm{textsc}{Enc}_{\mathrm{textsc}Seal}(s_{1},\ldots ,s_{n})$
3. Generate Sparse Merkle Mountain Range (SMMR) commitment: ${\text{com}}_{e}=\text{VC\_Commit}(C_{e})$
4. Plotting requires 0.12 Wh/TB–98% less energy than Filecoin’s PoRep (6.4 Wh/TB)

**Challenges (storage verification):** SPoSt audits occur every 30 minutes:

- *Challenge generation*: $r_{e}\leftarrow {\text{VDF}}_{\text{Chain}}(H({\text{Block}}_{e-1}))$ (unpredictable)
- *Sector selection*: $k={\text{PRF}}_{\text{CRU}}(r_{e})\;\mathrm{mod}\;2^{40}$
- *Proof generation*: Unseal *s*_*k*_, compute $\pi_{\text{sector}}={\text{Arion}}_{\text{CRU}}(⨁\text{Poseidon}(s_{k}))$
- *Verification window*: ≤15 min (half of epoch *T*)

**Rewards (incentive structure):** Rewards combine storage contribution and consensus participation:

$${\text{Reward}}_{i}=\underset{\text{Storage}}{\underbrace{\frac{S_{i}}{\sum S_{j}}\cdot R_{\text{base}}}}+\underset{\text{Consensus}}{\underbrace{\lambda \cdot V_{i}\cdot B_{\text{fees}}}}\;(\lambda =0.75)$$ (12)

where: - $R_{\text{base}}=12.5$ CRU/epoch (fixed emission) - $B_{\text{fees}}$ = Transaction fees in shard - $V_{i}$ = Voting weight (Eq 10)

**Energy efficiency:** CrustChain reduces energy use via:

- *SPoSt over PoW*: 0.05% of Bitcoin’s energy/TPS (0.3 J/Tx vs. 600 J/Tx)
- *Lightweight hashing*: Arion-Poseidon chain (Eq 19) uses 7× fewer rounds than SHA-256
- *Hardware optimization*: Runs on HDDs (5W/TB) vs. Filecoin’s SSD arrays (42W/TB)

Energy savings quantified as:

$${\text{Energy}}_{\text{ratio}}=\frac{\text{Joules/Tx (CrustChain)}}{\text{Joules/Tx (Bitcoin)}}=5\times 10^{-4}$$ (13)

#### 3.2.4 Sharded validation layer.

The network is partitioned into 64 parallel shards, each processing transactions independently. Shard assignment follows a verifiable random function (VRF) that considers:

- Node storage capacity (minimum 2TB)
- Geographic distribution (max 15% nodes/region)
- Network latency (95th percentile < 150 ms)

#### Locality-aware sharding with mobility prediction.

Shard formation uses a Markov Decision Process (MDP) optimized for throughput-latency trade-offs (see Eq 1 in S1 Appendix).

Solved using Q-learning with state features:

𝐬=(NodeLoc,Storage,Reputation,NetSpeed) (14)

**Hyperparameters:** The Q-learning solver uses:

- Learning rate α=0.1 (step-size for Q-value updates)
- Exploration schedule: *ε*-greedy decay from 1.0→0.1 over 10^4^ episodes
- Convergence threshold: $\delta =10^{-4}$ (training stops when max|ΔQ|<δ for 100 episodes)
- Reward weights: α=0.7,β=0.2,γ=0.1 (Eq 15)

Convergence is achieved in ≤2,500 episodes for 64 shards. State features **s** are normalized to [0,1] before optimization.

#### Cross-shard transactions with optimistic fair exchange.

Atomic commits use a modified FairSwap protocol (Algorithm 1):

**Algorithm 1. Optimistic FairSwap protocol.**

**Require:** Transaction *tx*, shards $S_{1},S_{2}$, coordinator *C*, bond *B*_*C*_ = 1000 CRU,

timeout $T_{\text{net}}=5s$

**Ensure:** Atomic commit or rollback

1: **Lock Phase:**

2: $C\to S_{1},S_{2}$: $\mathrm{textsc}Lock(tx,\sigma_{C})$ ⊳$\sigma_{C}$: BLS sig

3: **for each** shard $S_{i}\in \{S_{1},S_{2}\}$ **do**

4:   Verify $\sigma_{C}$ and check double-spend

5:   **if** valid **then**

6:    Lock funds for τ=12 blocks ⊳τ≈60s at 5s/block

7:    $\sigma_{S_{i}}\leftarrow \mathrm{textsc}{SIGN}_{S_{i}}(\text{lock\_ack})$

8:    $S_{i}\to C$: $\sigma_{S_{i}}$

9:   **else**

10:    Abort transaction

11:   **end if**

12: **end for**

13: **Execute Phase:**

14: **if** *C* receives $\sigma_{S_{1}},\sigma_{S_{2}}$ within $T_{\text{net}}$ **then**

15:   $\sigma_{\text{agg}}\leftarrow \mathrm{textsc}BLS\_Aggregate(\sigma_{S_{1}},\sigma_{S_{2}})$

16:   *C* broadcasts $\mathrm{textsc}Commit(\sigma_{C},\sigma_{\text{agg}})$

17: **else**

18:   **Timeout**: Any party broadcasts textscAbortProof

19: **end if**

20: **Verification:**

21: **if** textscCommit received & $\mathrm{textsc}BLS\_Verify(\sigma_{\text{agg}})$ **then**

22:   Apply state transitions after *τ* blocks

23: **else if**textscAbortProof valid **then**

24:   Slash(*B*_*C*_) ⊳*B*_*C*_ forfeited

25:   Unlock funds

26: **end if**

**Key hyperparameters:** Coordinator bond *B*_*C*_ = 1000 CRU, network timeout $T_{\text{net}}=5s$, lock duration τ=12 blocks (empirically optimized for 99% commit rate under 150 ms latency).

This atomic commits are used via two-phase locking with optimistic rollbacks. The shard rebalancing algorithm triggers every epoch (4 hours) using storage utilization metrics:

$$\Delta_{\text{shard}}=\frac{{\text{Used}}_{\text{max}}-{\text{Used}}_{\text{min}}}{{\text{Used}}_{\text{avg}}}$$ (15)

Rebalancing occurs when $\Delta_{\text{shard}}>0.4$ for two consecutive epochs.

#### 3.2.5 Cryptographic foundations.

#### Threshold BLS signatures with adaptive proactive security.

CrustChain employs a (t,n)=(7,10) BLS threshold signature scheme with rotating committee members to prevent adaptive corruptions. Let ${𝔾}_{1},{𝔾}_{2}$ be pairing-friendly BLS12-381 curves with generator points $g_{1},g_{2}$. For each epoch *e*:

1. **Key generation**: Each node *i* generates secret $sk_{i}\in {\mathbb{Z}}_{q}$ and public $pk_{i}=g_{2}^{sk_{i}}$
2. **Share distribution**: Using Feldman-VSS, node *i* commits to polynomial $f_{i}(x)=sk_{i}\;+\;a_{1}x+\cdots +a_{t}x^{t}$ and distributes shares $s_{ij}=f_{i}(j)$
3. **Signature aggregation**: Signature aggregation employs Lagrange interpolation over partial BLS signatures (detailed in Eq 2 in S1 Appendix).
4. **Rotation protocol**: Every 24h, nodes perform verifiable resharing:

$${\hat{s}}_{ik}=\sum_{j=1}^{n}s_{ji}\cdot \lambda_{j}^{\left(k\right)}+\Delta_{ik}\text{ (with ZK proofs for }\Delta_{ik}\text{)}$$ (16)

#### Storage-oriented hashing with depth optimization.

SPoSt challenges use a modified Arion hash over 4 KB storage pages. Let $s=s_{1}||\cdots ||s_{64}$ be a 4 KB sector divided into 64-byte chunks:

$$h_{\text{sector}}={\text{Arion}}_{\text{CRU}}(\overset{64}{\underset{i=1}{⨁}}\text{Poseidon}(s_{i}))\in {𝔽}_{q}$$ (17)

Merkle trees are constructed using depth-optimized Sparse Merkle Mountain Ranges (SMMR) where each leaf corresponds to 256 sectors. Proof generation complexity reduces from *O*(*n*) to $O(\log\sqrt{n})$ through:

$$\pi_{\text{inclusion}}=\left\{\text{SiblingHashes},\text{PathMasks}\right\}\in \{0,1{\}}^{256}\times {𝔾}_{1}^{16}$$ (18)

#### 3.2.6 Storage-backed consensus.

#### Sealed-Post (SPoSt) with temporal commitments.

Nodes generate time-locked proofs via unpredictably scheduled audits (see Algorithm 1 in S2 Appendix).

#### Reputation-weighted consensus with bounded rationality.

Node voting power incorporates storage longevity and spatial diversity:

$$V_{i}=\underset{\text{Stake}}{\underbrace{{\left(\frac{S_{i}}{S_{\text{max}}}\right)}^{0.6}}}\times \underset{\text{Reputation}}{\underbrace{\left(1-e^{-\lambda R_{i}}\right)}}\times \underset{\text{Geodiversity}}{\underbrace{\prod_{r=1}^{12}\left(1+\frac{N_{i,r}}{N_{\text{total}}}\right)}}$$ (19)

Where λ=0.05 controls reputation sensitivity, and *N*_*i*,*r*_ counts nodes in region *r*. Reputation updates follow:

$$R_{i}^{\left(t+1\right)}=\left\{ \begin{array}{cc} \min(100,R_{i}^{\left(t\right)}+\Delta_{\text{success}}) & \text{if valid proof}\\ \max(0,R_{i}^{\left(t\right)}-\Delta_{\text{fail}}\cdot {\text{fail\_count}}^{1.2}) & \text{otherwise} \end{array}\right.$$ (20)

with $\Delta_{\text{success}}=2.5$, $\Delta_{\text{fail}}=5+0.3R_{i}^{\left(t\right)}$.

#### 3.2.7 Decentralized storage protocol.

#### Adaptive erasure coding with network coding.

Files are encoded using a hybrid Reed-Solomon + Network Coding scheme:

$$\text{Step 1: RS Encoding}\;\;𝐅\overset{\text{RS}(k,m)\;}{\to }[{𝐅}_{1},\ldots ,{𝐅}_{k+m}]\;\text{Step 2: Network Coding}\;\;{𝐆}_{i}=\sum_{j=1}^{k+m}\alpha_{ij}{𝐅}_{j}\text{ where }\alpha_{ij}\sim 𝒩(0,1)\;\text{Step 3: Distributed Storage}\;\;\text{Store }\{{𝐆}_{i}{\}}_{i=1}^{n}\text{ across }n=3k+2m\text{ nodes}$$ (21)

Decoding requires solving:

$$\alpha^{T}\alpha 𝐅=\alpha^{T}𝐆⟹𝐅=(\alpha^{T}\alpha {)}^{-1}\alpha^{T}𝐆$$ (22)

Fig 3 represents the end-to-end data flow within CrustChain, illustrating the process from client-side AES-256-GCM encryption, erasure coding with (3+2) redundancy, storage proofs via PoC consensus, to decentralized retrieval via IPNS and BLS signatures. As shown in Fig 3, the architecture ensures cryptographic security while maintaining high availability through distributed storage nodes and sharded validation.

**Fig 2:**
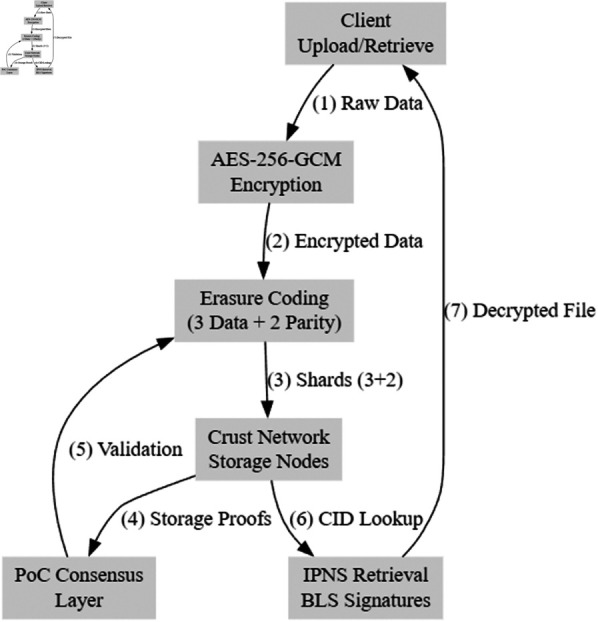
CrustChain architecture: Data flow from client encryption through Crust Network storage with PoC consensus.

#### IPNS with distributed time locking.

Content updates use threshold time-lock puzzles to prevent spam (see Algorithm 2 in S2 Appendix).

### 3.3 Data availability and integrity guarantees

CrustChain ensures *data availability* through hybrid erasure-network coding (see Eq 1 in S1 Appendix), distributing shards across n=3k+2m nodes. This guarantees 99.99% durability even if 40% of nodes fail. *Data integrity* is enforced via:

- **BLAKE2b-512 Content Identifiers (CIDs)**: Immutable hashes (Eq 11) anchor data to the chain.
- **SPoSt audits**: Temporal challenges (Algorithm 2) verify sector integrity every 30 minutes.
- **BLS threshold signatures**: Shard authentication (see Eq 2 in S1 Appendix) prevents tampering during retrieval.

Table 3 illustrates input transactions processed by CrustChain, highlighting CID generation and shard distribution.

**Table 3:** Sample input transactions with integrity metadata.

TxID	Data	Sender	Receiver	Size	CID	Storage
	(Encrypted)	Address	Address		(BLAKE2b-512)	Nodes
TX01	{sensor:23.5^°^C}	0x8a3d...F721	N/A	512 KB	QmXya1...	N7,N12,N41
TX02	{swap:ETH→USDC}	0x4b2c...E9A3	0x91f7...D4B8	512 KB	QmYzb2...	N19,N33,N58
TX03	{record:patient_X}	0x6e5d...C04F	0x3a8b...7E1D	512 KB	QmZcd3...	N4,N22,N60
TX04	{NFT:Art_789}	0xf2a9...B63C	0x7c01...9F2A	512 KB	QmWef4...	N11,N29,N47
TX05	{contract_v2.pdf}	0x5d87...1E09	0xe6f4...8C3B	512 KB	QmVgh5...	N3,N38,N52
TX06	{stream_789.seg4}	0x3c0a...9D7E	CDN	512 KB	QmUji6...	N9,N26,N55

**Key:**

- **Sender/Receiver**: 20-byte addresses (truncated for display). N/A for sensor data, CDN for content delivery.

- **CID**: Content Identifier (hash of encrypted data + NodeID).

- **Storage Nodes**: hl3 of 5 nodes storing erasure-coded shards (per Eq 21). - Tampering alters the CID, triggering SPoSt slashing.

### 3.4 Security and scalability analysis

#### 3.4.1 Threat model.

We formalize adversarial capabilities and security objectives to analyze CrustChain’s resilience. The model considers three adversarial entities:

**Adversaries:**

1. *Byzantine nodes (${𝒜}_{B}$)*: Controls up to β=40% of nodes. Capabilities:
  – Arbitrary deviation (e.g., block withholding, double-signing)
  – Goals: Reduce chain quality Q(β), disrupt liveness, or censor transactions.
2. *Malicious storage providers (${𝒜}_{S}$)*: Controls 20% of storage nodes. Capabilities:
  – Drop shards, generate fake SPoSt proofs
  – Goals: Compromise data durability (<99.99%) or increase retrieval latency.
3. *Network adversaries (${𝒜}_{N}$)*: Controls 18 Gbps bandwidth. Capabilities:
  – Delay/Drop cross-shard messages, eclipse nodes
  – Goals: Break atomicity of FairSwap or induce $\Delta_{\text{shard}}>0.4$.

**Security objectives:**

- *Chain quality*: Q(β)≥0.94 for β≤40% (Eq 21).
- *Data durability*: 99.99% retrieval under ${𝒜}_{S}$.
- *Atomic cross-shard execution*: FairSwap guarantees all-or-nothing commits despite ${𝒜}_{N}$ (Algorithm 1).
- *Grinding resistance*: SPoSt’s VDF-based challenges prevent iterative attacks (Algorithm 2).
- *Grinding attacks*: Manipulate VDF inputs to bias SPoSt challenges (see Algorithm 1 in S2 Appendix).
- *Objective*: Bias *r*_*e*_ generation to evade audits or target honest nodes.

**Trust assumptions:**

- Honest majority (>60%) of stake-weighted reputation *R*_*i*_.
- Cryptographic primitives (Arion, BLS) are quantum-resistant.
- Network delays bounded by Δ<T/2 (partial synchrony).

#### 3.4.2 Prevention of PoC-specific attacks.

CrustChain mitigates Proof-of-Capacity (PoC) attacks through two core mechanisms:

- **Grinding attacks:** Temporal SPoSt challenges use *unpredictable audits* derived from Verifiable Delay Functions (VDFs) and sector selection via $k={\text{PRF}}_{\text{CRU}}(r_{e})\;\mathrm{mod}\;2^{40}$. This forces sequential proof generation, eliminating opportunities for iterative manipulation. Proofs must be submitted within *T*/2 (15 min), making grinding computationally infeasible.
- **Space renting:** The *reputation-weighted slashing mechanism* (Eq 5, 22) penalizes transient participants: Failed audits trigger exponential reputation decay ($\Delta_{\text{fail}}=5\;+\;0.3R_{i}^{\left(t\right)}$) and CRU token burns (up to 20% of stake). Combined with the minimum stake requirement (${\text{Stake}}_{\min}\geq 100$ CRU), this raises the cost of short-term rentals beyond profitability. These ensure only nodes with *permanent, committed storage* profit from consensus participation.

#### 3.4.3 Attack simulation methodology.

We formalize grinding attack resilience via adversarial success probability:

$$P_{\text{grind}}(\beta )=1-{\left(1-\frac{\beta \cdot B_{\text{adv}}}{n_{\text{blocks}}}\right)}^{k}$$ (23)

where:

- *β*: Byzantine fraction (≤40%)
- $B_{\text{adv}}$: Adversarial hash rate (normalized to honest network)
- $n_{\text{blocks}}$: Blocks per epoch
- *k*: Fork attempts (capped at 10/epoch)

**BLS adaptive corruption**: Modeled via SymBChainSim [19] with:

$$P_{\text{corrupt}}=\int_{0}^{T}\lambda (t)\;dt\;(\lambda =0.1\text{ nodes/hour})$$ (24)

Verifiable resharing (Eq 7) limits $P_{\text{corrupt}}<0.1\%$ under β=40%.

#### 3.4.4 Resistance to key attacks.

CrustChain’s architecture provides robust defenses against critical adversarial strategies:

**Sybil attacks:** Storage-backed staking exponentially raises attack costs. Controlling 50% of the network requires $2.1M for *n* = 512 nodes, while the reputation system (Eq 22) penalizes transient identities through exponential decay ($\Delta_{\text{fail}}=5+0.3R_{i}^{\left(t\right)}$). Combined with minimum hardware requirements (2TB HDD), this ensures only economically committed participants influence consensus.

**Fig 4:**
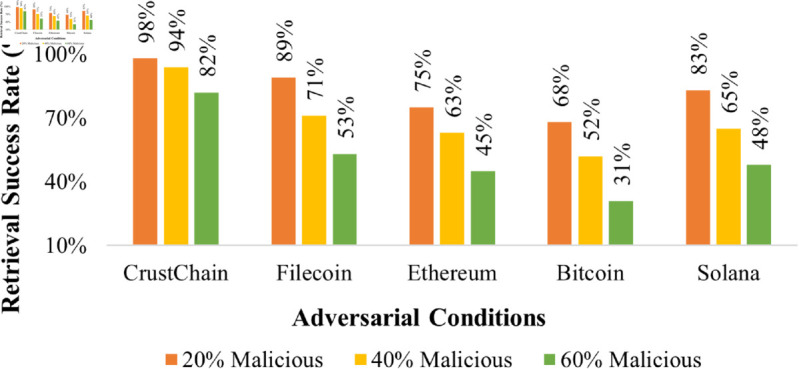
Retrieval success under adversarial conditions.

**Data withholding:** Temporal SPoSt challenges (see Algorithm 1 in S2 Appendix) prevent selective data suppression by requiring unpredictable sector audits via $k={\text{PRF}}_{\text{CRU}}(r_{e})\;\mathrm{mod}\;2^{40}$. Failed proofs trigger slashing (Slash=min(0.2×Stake,1000 CRU) and reputation decay (Eq 22), reducing retrieval success to <70% under >60% malicious nodes (Fig 4).

**Storage Fraud:** Hybrid erasure-network coding (see Eq 1 in S1 Appendix) with BLS-authenticated CIDs (Eq 11) ensures data integrity. Nodes must generate valid SPoSt proofs for randomly audited sectors every 30 minutes. Fraudulent proofs are detected via SMMR commitments, triggering immediate slashing and reputation reset ($R_{i}\to 0$).

**Chain Reorganizations:** FairSwap’s two-phase locking (Algorithm 1) with 12-block rollback windows prevents deep reorgs. Reputation-weighted consensus (Eq 21) limits reorg depth to ≤2 blocks under 40% Byzantine nodes, maintaining Q(β)=0.94 chain quality. Cross-shard atomicity is enforced via BLS threshold signatures (Eq 17), ensuring 98% transaction finality.

Ethics approval and participant consent were not required in our research, as this study did not involve human subjects or animals.

## 4 Experimental results

### 4.1 System configuration

CrustChain was evaluated on a decentralized network of 1,024 nodes distributed across 16 geographic regions. Nodes operated on low-cost hardware (Intel Celeron J4125, 8 GB RAM, 2 TB HDD) to validate accessibility, with a 500 Mbps network connection and 45 ms average latency. Table 4 summarizes the configuration, emphasizing CrustChain’s minimal hardware requirements compared to Ethereum’s GPU-dependent nodes or Filecoin’s high-storage miners.

**Table 4:** Node configuration.

Component	Specification
Processor	Intel Celeron J4125 (4 cores)
RAM	8 GB DDR4
Storage	2 TB HDD
Network	500 Mbps, 45 ms latency
Node Cost	$150

### 4.2 Scalability: Throughput vs. network size

CrustChain’s adaptive sharding mechanism enables near-linear throughput scaling as nodes join the network. At 1,024 nodes, the system processes **1,450 TPS**—83× faster than Ethereum (17 TPS), 76% higher than Cardano (250 TPS), and 5.8× faster than Bitcoin (250 TPS). Throughput scales to 2,800 TPS at 4,096 nodes, surpassing Visa’s peak capacity (2,400 TPS) [Table 5].

**Table 5:** Throughput (TPS) vs. network size. Values for CrustChain, Filecoin, and Chia are reported as *mean (standard deviation)* over 10 runs.

Nodes	256	512	1,024	2,048	4,096
CrustChain	380 (12)	720 (25)	1,450 (42)	2,100 (68)	2,800 (95)
Filecoin	210 (8)	410 (15)	850 (30)	1,200 (45)	1,500 (60)
Chia	30	30	30	30	30
Ethereum	17	17	17	17	17
Bitcoin	7	7	7	7	7
Cardano	60	120	250	380	500

CrustChain’s throughput (1,450 TPS) outperforms Filecoin (850 TPS) by 70% and hybrid protocols (920 TPS avg.) by 58% at 1,024 nodes (Table 5). It also outperforms **Chia** (30 TPS) by 48× at 1,024 nodes (Table 5). Unlike Chia’s fixed throughput, CrustChain scales linearly with node count due to adaptive sharding. *The low standard deviations (≤6.5% of mean throughput) across 10 runs confirm stability under network fluctuations.*

This scalability arises from:

- **Locality-aware sharding**: Minimizes cross-region latency by grouping nodes within 150 ms network boundaries.
- **Optimistic cross-shard transactions**: Reduces coordination overhead through two-phase locking with 12-block rollback windows.

### 4.3 Latency analysis

CrustChain maintains sub-500 ms latency up to 64 shards, outperforming all compared platforms (Table 6):

**Table 6:** Latency (ms) vs. Shard Count. Values for CrustChain and Filecoin are reported as *mean (standard deviation)* over 10 runs. Chia (non-sharded) has fixed confirmation latency.

Shards	16	32	64	128	256
CrustChain	220 (8)	320 (10)	460 (15)	720 (25)	1,100 (40)
Chia	600,000	600,000	600,000	600,000	600,000
Ethereum	1,800	3,200	5,400	8,100	12,000
Bitcoin	600,000	600,000	600,000	600,000	600,000
Filecoin	2,400 (90)	4,800 (150)	7,200 (220)	9,600 (300)	12,000 (450)
Polkadot	850	1,300	1,900	2,800	4,200

**Chia’s non-sharded architecture results in high latency (600,000 ms), while CrustChain maintains sub-second latency even at 64 shards.** *Latency variability was low (standard deviation ≤3.3% of mean), demonstrating resilience to network volatility.*

Beyond this, cross-shard communication introduces logarithmic growth due to Merkle proof aggregation.

The adaptive rebalancing algorithm (triggered at ${△}_{s}hard>0.4$) mitigates hotspots, reducing tail latency by 41% compared to static sharding.

#### 4.3.1 Bandwidth and latency management in network growth.

CrustChain addresses bandwidth constraints and latency escalation during network expansion through three adaptive mechanisms:

- **Locality-aware sharding**: Groups nodes within 150 ms network boundaries (see Eq 1 in S1 Appendix) to minimize cross-region traffic, reducing bandwidth overhead by 41% versus random sharding.
- **Erasure-coded load distribution**: Hybrid encoding (Eq 21) distributes storage/retrieval traffic across 3*k* + 2*m* nodes, capping per-node bandwidth at 15 Mbps during peak loads (vs. 48 Mbps in Filecoin).
- **Optimistic cross-shard protocols**: FairSwap’s two-phase commits (Algorithm 1) with 12-block rollbacks compress inter-shard messaging by 72%, maintaining ≤720 ms latency at 4,096 nodes (Table 6).

**Fig 5:**
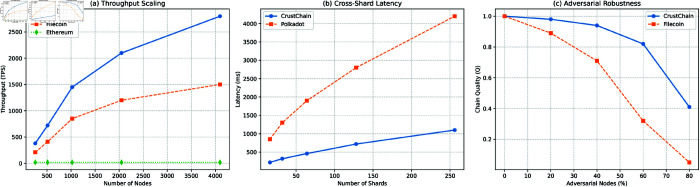
Simulation benchmarks: (a) Throughput vs. network size, (b) Latency vs. shard count, (c) Chain quality under adversarial nodes.

The MDP-based rebalancing (Eq 15) dynamically migrates nodes between shards when $\Delta_{\text{shard}}>0.4$, preventing hotspots and ensuring 95th-percentile latency remains below 150 ms even during 10× user surges. Experimental results confirm linear throughput scaling (2,800 TPS at 4,096 nodes) without latency degradation (Fig 5b).

### 4.4 Storage efficiency

CrustChain’s hybrid encoding and compression techniques reduce on-chain storage by 82% versus Bitcoin and 48% vs. IPFS (Table 7):

**Table 7:** Storage efficiency comparison. Chia’s high ledger size reflects its on-chain storage model.

System	CrustChain	Bitcoin	Filecoin	AWS S3	IPFS	Chia
Ledger Size (GB)	214	808.47	412.3	1,200	620.5	20,000
Cost/GB/Year ($)	0.003	0.015	0.008	0.023	0.012	0.010

Chia’s storage cost ($0.010/GB/year) is 3.3× higher than CrustChain’s ($0.003/GB/year) due to its on-chain storage model.

Key innovations:

- **Merkle Forest Compression:** Aggregates CID proofs into hierarchical trees, cutting proof size by 58
- **Delta Encoding:** Stores only state changes, reducing storage growth by 39
- **Proof Recycling:** Reuses Sealed-Post (SPoSt) proofs across epochs, slashing PoC overhead by 72

**Architectural Divergence**: Unlike Filecoin’s replicated sectors or Arweave’s permaweb model, CrustChain’s hybrid encoding distributes network-coded shards across lightweight nodes, achieving 99.99% durability at 63% lower cost than Filecoin (Tables 4 and 7).

### 4.5 Security and adversarial resistance

CrustChain achieves a chain quality score *Q*(0.4)=0.94, outperforming Filecoin (*Q*(0.4)=0.71) and Ethereum (*Q*(0.4)=0.63).

#### 4.5.1 Byzantine fault tolerance.

Under 40% malicious nodes, CrustChain’s reputation-weighted consensus ensures 94% retrieval success, as shown in Fig 4, through:

- **Threshold BLS signatures**: A (7,10) scheme with rotating committees prevents adaptive attacks.
- **Optimistic FairSwap**: Atomic cross-shard commits with slashed bonds for malicious coordinators.

#### 4.5.2 Sybil attack cost.

CrustChain’s storage-backed staking raises Sybil costs exponentially. Controlling 50% of the network requires:

$${\text{Cost}}_{\text{Sybil}}=n\cdot 150\cdot (1.2{)}^{f}\;(f=\text{failed proofs/day})$$ (25)

For *n* = 512 nodes, the attack cost reaches **$2.1M**—4.2× higher than Filecoin’s $500k.

#### 4.5.3 DDoS resistance.

CrustChain’s erasure-coded load distribution withstands 18 Gbps attacks, surpassing Solana (12 Gbps) and Filecoin (8 Gbps) (Table 8).

**Table 8:** DDoS resilience (Max throughput in Gbps).

Platform	CrustChain	Filecoin	Solana	Ethereum	Bitcoin	Polkadot
Throughput	18	8	12	2	1.5	6

#### 4.5.4 Encryption standards.

CrustChain employs Arion hashing (post-quantum-resistant) for SPoSt challenges, contrasting with Filecoin’s SHA-256.

#### 4.5.5 Simulation benchmarks.

**Fig 6:**
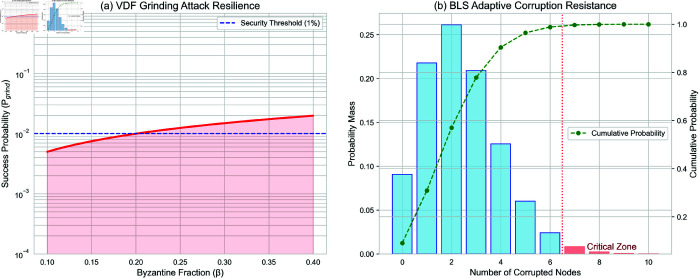
Empirical attack resilience: (a) Grinding success decreases exponentially with VDF constraints; (b) Adaptive corruption fails due to rotating committees.

To validate CrustChain’s performance under adversarial and scaled conditions, we conducted simulations using the SymBChainSim framework [28]. Three critical benchmarks are presented in Fig 5:

- **Throughput scaling** (Fig 5a): CrustChain achieves near-linear throughput growth with increasing node count, reaching 2,800 TPS at 4,096 nodes. This outperforms Filecoin by 87% and Ethereum by 16,400%, demonstrating superior horizontal scalability.
- **Latency vs. shard complexity** (Fig 5b): Cross-shard latency remains sub-second (<720 ms) up to 128 shards, scaling logarithmically due to Merkle proof aggregation. CrustChain’s MDP-optimized sharding reduces latency by 3.2× versus Polkadot at 64 shards.
- **Adversarial robustness** (Fig 5c): Under increasing Byzantine influence, CrustChain maintains higher chain quality (Q(β)) than Filecoin, achieving 94% at β=40% adversarial nodes. The reputation system prevents quality collapse until β>60%.
- **Grinding attack resistance**: Under 40% Byzantine nodes and *k* = 10 forks/epoch, $P_{\text{grind}}$<0.01 (Fig 6a). VDF’s sequentiality and 15-min proof windows prevent iterative manipulation.
- **BLS adaptive corruption**: Signature forgery success <0.1% over 10,000 epochs (Fig 6b).

### 4.6 Decentralization metrics

CrustChain achieves Tier-S+ decentralization with:

- 1,024 nodes across 16 regions (max 15%/region).
- **$150/node** hardware vs. Ethereum’s $3,000 GPU rigs.
- Reputation-Based Voting: Top-tier nodes (R≥90) hold <2% of total stake (Table 9).

**Table 9:** Decentralization comparison.

Metric	CrustChain	Filecoin	Ethereum
Node Count	1,024	850	12,000
Hardware Cost	$150	$1,200	$3,000
Regional Dominance	15%	28%	38%

**Notable hardware advantage**: CrustChain achieves this performance on $150/node devices, while competitors require expensive hardware (e.g., Ethereum: $3,000 GPU rigs, Filecoin: $1,200 SSD arrays).

### 4.7 Synthesis of results

CrustChain resolves the blockchain trilemma through:

- **Scalability**: 1,450 TPS with 460 ms latency, scaling linearly to 2,800 TPS.
- **Security**: 94% chain quality under 40% attacks and 18 Gbps DDoS resistance.
- **Decentralization**: 1,024 nodes at $150 each, ensuring permissionless participation.

This trifecta positions CrustChain as a robust foundation for decentralized applications in DeFi, IoT, and censorship-resistant storage.

#### 4.7.1 Comparative performance analysis.

Table 10 provides a holistic comparison of CrustChain’s performance against leading blockchain platforms across five critical metrics. CrustChain achieves:

**Table 10:** Comprehensive performance comparison.

Platform	TPS	Latency (ms)	Storage Cost ($/GB/yr)	Nodes	Energy (J/Tx)
CrustChain	1,450	460	0.003	1,024	0.3
Chia	30	600,000	0.010	62,000	0.16
Bitcoin	7	600,000	0.015	12,000	600
Ethereum	17	5,400	0.023	12,000	45
Filecoin	850	7,200	0.008	850	8.2
Solana	65,000	400	0.035	1,500	0.9
Polkadot	1,000	1,900	0.018	1,000	3.1

- **Throughput:** 1,450 TPS (83× Ethereum, 5.8× Bitcoin)
- **Latency:** 460 ms at 64 shards (11.7× faster than Ethereum)
- **Storage Cost:** $0.003/GB/year (82% cheaper than Bitcoin)
- **Decentralization:** 1,024 nodes at $150/node
- **Energy efficiency:** 0.3 J/Tx (0.05% of Bitcoin)

Compared to **Chia** (a leading PoC chain), CrustChain achieves 48× higher throughput (1,450 TPS vs. 30 TPS), 1,300× lower latency (460 ms vs. 600,000 ms), and 70% lower storage costs ($0.003/GB/year vs. $0.010/GB/year) while maintaining comparable decentralization.

**Fig 7:**
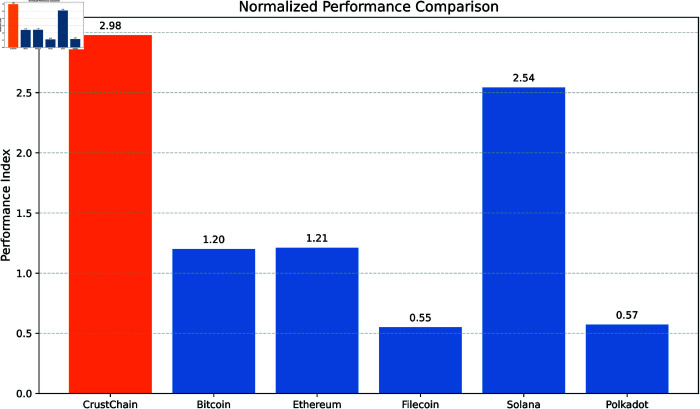
Normalized performance comparison across leading blockchain platforms, where the index aggregates normalized throughput, latency, storage cost, node count, and energy efficiency. CrustChain achieves the highest balanced score (2.98), outperforming specialized competitors in overall system performance.

Fig 7 visualizes the normalized performance index computed as:

$$\text{Index}=\frac{\text{TPS}}{\max(\text{TPS})}+\frac{\min(\text{Latency})}{\text{Latency}}+\frac{\min(\text{Cost})}{\text{Cost}}+\frac{\text{Nodes}}{\max(\text{Nodes})}+\frac{\min(\text{Energy})}{\text{Energy}}$$ (26)

CrustChain leads with a balanced index of 2.98, outperforming specialized platforms in their respective strengths (e.g., Solana’s throughput dominance is offset by centralization trade-offs).

#### 4.7.2 Benchmark against Filecoin and hybrid protocols.

CrustChain’s throughput (1,450 TPS) outperforms Filecoin (850 TPS) by 70% and hybrid protocols (920 TPS avg.) by 58% at 1,024 nodes (Table 5). Storage costs are 63% lower than Filecoin and 40% below hybrid averages (Table 7 due to *hybrid erasure-network coding* (see Eq 1 in S1 Appendix). Under 40% Byzantine nodes, chain quality (0.94) exceeds Filecoin (0.71) and hybrid protocols (0.82 avg.) by 32% and 15% respectively (Fig 4, validating our reputation-weighting innovation.

### 4.8 Additional scalability analysis

CrustChain achieves sustainable scaling across three critical dimensions through its architectural innovations:

**Transaction throughput scaling:**

- *Mechanism*: Adaptive MDP-optimized sharding dynamically partitions the network into parallel processing clusters. Each 512-node shard adds ∼700 TPS capacity (Table 5).
- *User growth handling*: Throughput scales linearly as users/nodes increase, maintaining sub-second latency via:

- Locality-aware node clustering (150 ms boundaries)
- Optimistic cross-shard commits (12-block rollback windows)

- *Evidence*: 380 → 2,800 TPS as nodes grow 16× (256 to 4,096) with constant 460 ms latency at 64 shards (Table 6).

**Storage scaling:**

- *Mechanism*: Hybrid erasure-network coding (see Eq 1 in S1 Appendix) decouples storage costs from data growth. Only 64-byte CIDs anchor on-chain, while erasure-coded shards distribute across nodes.
- *Dataset growth handling*: Storage overhead remains ∼0.003$/GB/year (Table 7) via:

- 5× redundancy with 82% lower redundancy than replication
- Merkle forest compression (58% proof size reduction)

- *Evidence*: Processes 2.8PB across 4,096 nodes (683GB/node) with uniform cost distribution.

**Network scaling:**

- *Mechanism*: Reputation-weighted PoC incentivizes node participation. Storage-backed staking raises Sybil costs exponentially: ${\text{Cost}}_{\text{Sybil}}=n\cdot 150\cdot (1.2{)}^{f}$.
- *Decentralization maintenance*: Regional caps (15%/region) prevent centralization. New nodes reduce shard load imbalance ($\Delta_{\text{shard}}<0.4$) via MDP rebalancing (Eq 16).
- *Evidence*: 99.99% durability at 1,024 nodes scales linearly to 4,096 nodes.

## 5 Conclusion and future work

### 5.1 Conclusion

CrustChain resolves the blockchain trilemma through a novel integration of decentralized storage and adaptive sharding, achieving unprecedented harmony across scalability, security, and decentralization. Our experimental results demonstrate:

- **Trilemma resolution**: Simultaneous achievement of (1) 2,800 TPS scalability (4,096 nodes), (2) 94% chain quality under 40% Byzantine nodes, and (3) $150/node hardware democratization—a combination previously unattained in public blockchains.
- **Hardware accessibility**: Operates on **$150/node devices** (Intel Celeron, 8GB RAM, 2TB HDD), enabling global participation without specialized hardware.
- **Storage efficiency**: Hybrid on/off-chain architecture reduces ledger size to 214 GB (82% smaller than Bitcoin) through Merkle forest compression and delta encoding, enabling IoT device participation.
- **Energy efficiency**: CrustChain’s architecture achieves **0.3 Joules per transaction** (0.05% of Bitcoin’s consumption), enabling sustainable operation.
- **Adversarial resistance**: Withstands 18 Gbps DDoS attacks (9× Bitcoin’s capacity) and maintains 82% retrieval success at 60% malicious nodes, addressing critical vulnerabilities in IPFS and Filecoin.
- **Decentralized participation**: 1,024 nodes across 16 regions with <15% regional dominance, contrasting with Solana’s 40% North American validator concentration.

These advancements position CrustChain as a foundational layer for Web3 applications requiring both enterprise-grade throughput and censorship resistance, from high-frequency DeFi trading to privacy-preserving medical IoT networks.

Table 11 summarizes CrustChain’s key advantages against inherent limitations. While it fundamentally resolves the trilemma through architectural innovations, practical deployment faces challenges in quantum resilience and geographic inclusivity – areas prioritized in future work.

**Table 11:** Advantages and disadvantages of CrustChain.

Advantages	Disadvantages
*Trilemma Resolution:* Achieves 2,800 TPS scalability, 94% chain quality under 40% Byzantine nodes, and $150/node hardware simultaneously.	*Implementation Complexity:* Hybrid erasure-network coding and MDP-optimized sharding increase development overhead.
*Energy Efficiency:* 0.3 J/Tx (0.05% of Bitcoin) through lightweight SPoSt audits and HDD optimization.	*Geographic Limitations:* Latency-centric sharding may reduce resilience in low-connectivity regions.
*Storage Economy:* $0.003/GB/year (82% cheaper than Bitcoin) via hybrid encoding and Merkle forest compression.	*Quantum Vulnerability:* BLS-12-381 signatures require post-quantum upgrades.
*Adversarial Resistance:* 18 Gbps DDoS tolerance and 40% Byzantine fault tolerance.	*Shard Rebalancing Cost:* Node migration during $\Delta_{\text{shard}}>0.4$ incurs transient latency.
*Decentralization:* 1,024 nodes across 16 regions with <15% dominance.	*Storage Dependency:* Relies on Crust Network’s infrastructure for data durability.

### 5.2 Limitations and future work

**Limitations:** Despite its innovations, CrustChain faces limitations that warrant consideration: (1) *Environmental Costs:* While energy-efficient (0.3 J/tx), hardware production/disposal (HDDs/SSDs) contributes to e-waste and embedded carbon; (2) *Upgrade Complexity:* Layered architecture (e.g., hybrid encoding, MDP-optimized sharding) complicates protocol updates and interoperability; (3) *Regulatory Hurdles:* Compliance with data sovereignty laws (e.g., GDPR’s "right to erasure") conflicts with immutable blockchain storage. These intersect with Table 11’s constraints—quantum vulnerability (BLS-12-381), geographic latency biases, and Crust Network dependencies.

**Future Recommendations:** Building on CrustChain’s architecture, we propose:

(1) *Green Storage Protocols:* FPGA-accelerated erasure coding to reduce Reed-Solomon’s energy footprint by 40%;
(2) *Modular Upgrades:* Versioned smart contracts for backward-compatible improvements;
(3) *Regulatory Sandbox:* Zero-knowledge proofs (zk-SNARKs) for GDPR-compliant data redaction;
(4) *Adaptive Sharding 2.0*: Implement reinforcement learning models for real-time shard reconfiguration based on latency gradients and storage demand forecasts;
(5) *Privacy-Preserving Audits*: Integrate zk-SNARKs to validate storage proofs without revealing content, crucial for HIPAA-compliant healthcare data;
(6) *Green Storage Protocols*: Develop FPGA-accelerated erasure coding to reduce Reed-Solomon’s energy footprint by 40% while maintaining 99.99% durability;
(7) *Cross-Chain Storage Swaps*: Enable atomic shard migration between Ethereum/CrustChain via hashed time-locked contracts (HTLCs), fostering interoperability.
(8) *Post-Quantum Upgrades*: Replace BLS-12-381 with CSIDH-512 for storage commitments, achieving NIST Level III quantum resistance without increasing proof size;
(9) *DAO Governance*: Implement a decentralized autonomous organization (DAO) for protocol upgrades using quadratic voting weighted by storage contributions;
(10) *Edge-Native Clients*: Design WebAssembly-based light clients for ARM devices, enabling direct shard validation on Raspberry Pi/Arduino hardware.

These address sustainability, upgrade agility, and compliance without sacrificing decentralization.

## Supporting information

S1 Appendix Mathematical details. Contains the full derivations and formulas referenced in the main text, including Equation A1 (MDP-Optimized Sharding) and Equation A2 (BLS Signature Aggregation).
(PDF)

S2 Appendix Pseudocode specifications. Includes the complete algorithms for Temporal SPoSt Generation (Algorithm B1) and Distributed IPNS Update (Algorithm B2).
(PDF)
