# Supplementary material for: CrustChain: Resolving the blockchain trilemma via decentralized storage and proof-of-capacity consensus
Source: PLoS One. 2025 Aug 18;20(8):e0328395. doi: 10.1371/journal.pone.0328395 (PMC12360553; doi:10.1371/journal.pone.0328395)
Supplement: S1 Appendix — Contains the full derivations and formulas referenced in the main text, including Equation A1 (MDP-Optimized Sharding) and Equation A2 (BLS Signature Aggregation). (PDF) [file pone.0328395.s001.pdf]

S1 Appendix: Mathematical Details

Equation 1: MDP-Optimized Sharding

$$\max_{\pi} \mathbb{E}\left[\sum_{t=0}^{\infty} \gamma^t \left(\alpha B_t - \beta L_t - \gamma M_t\right)\right]$$

Where: - B_t: Block throughput - L_t: Cross-shard latency - M_t: Migration cost - $\gamma = 0.9$: Discount factor

Equation 2: BLS Signature Aggregation

For message m, partial signature $\sigma_j = H_{\mathbb{G}_1}(m)^{s_{ij}}$. Combine using:

$$\sigma = \prod_{j \in Q} \sigma_j^{\lambda_j} \text{ where } \lambda_j = \prod_{\substack{k \in Q \\ k \neq j}} \frac{k}{j - k}$$
