# Supplementary material for: CrustChain: Resolving the blockchain trilemma via decentralized storage and proof-of-capacity consensus
Source: PLoS One. 2025 Aug 18;20(8):e0328395. doi: 10.1371/journal.pone.0328395 (PMC12360553; doi:10.1371/journal.pone.0328395)
Supplement: S2 Appendix — Includes the complete algorithms for Temporal SPoSt Generation (Algorithm B1) and Distributed IPNS Update (Algorithm B2). (PDF) [file pone.0328395.s002.pdf]

S2 Appendix: Pseudocode Specifications

Algorithm 1

Algorithm 2 Temporal SPoSt Generation

Precompute (Epoch $e-1$):
  Store $C_e = \text{Enc}_{\text{SEAL}}(s_1, \ldots, s_n)$ using SGX sealing
  Generate commitment $\text{com}_e = \text{VC.Commit}(C_e)$
Challenge (Epoch e):
  Receive $r_e \leftarrow \text{VDF}_{\text{Chain}}(H(\text{Block}_{e-1}))$
  Compute $k = \text{PRF}_{\text{CRU}}(r_e) \mod 2^{40}$
  Unseal $s_k = \text{Dec}_{\text{SEAL}}(C_e, k)$
  Generate $\pi_{\text{sector}} = \text{SMMR.Prove}(s_k, \text{com}_e)$
  Submit π_{sector} before $t_{\text{epoch}} + T/2$
Slashing:
  If $\nexists \pi_{\text{sector}} \vee \text{Verify}(\pi_{\text{sector}}) = 0$:
    Slash $\min(0.2 \times \text{Stake}, 1000\ \text{CRU})$

Algorithm 2

Algorithm 3 Distributed IPNS Update

Input: New CID c_{new}, Seq number s
Generate puzzle $Z = \text{SHA3}(c_{\text{new}} \parallel s)$
Compute $T = \text{Puzzle}(Z, t)$ where $t = 2^{30}$ steps
Split T into $n = 5$ shards using VSS
Broadcast $(c_{\text{new}}, s, \{T_i\})$ to DHT
Validation:
  Wait until ≥ 3 shards received
  Reconstruct $T' = \sum \lambda_i T_i$
  Verify Puzzle.Verify(Z, T')
  If valid, update IPNS record
